# Study of Minor Chromophores in Biological Tissues by Diffuse Optical Spectroscopy (Review)

**DOI:** 10.17691/stm2025.17.1.12

**Published:** 2025-02-28

**Authors:** K.A. Bylinskaya, V.V. Perekatova, I.V. Turchin

**Affiliations:** Junior Researcher, Laboratory of Biophotonics; A.V. Gaponov-Grekhov Institute of Applied Physics of the Russian Academy of Sciences, 46 Ulyanov St., Nizhny Novgorod, 603950; PhD, Researcher, Laboratory of Biophotonics; A.V. Gaponov-Grekhov Institute of Applied Physics of the Russian Academy of Sciences, 46 Ulyanov St., Nizhny Novgorod, 603950; PhD, Head of the Department of Radiophysical Methods in Medicine; Head of the Laboratory of Biophotonics; A.V. Gaponov-Grekhov Institute of Applied Physics of the Russian Academy of Sciences, 46 Ulyanov St., Nizhny Novgorod, 603950

**Keywords:** diffuse optical spectroscopy, diffuse reflectance spectroscopy, chromophores, tissue optics, oxygenation, methemoglobin, carboxyhemoglobin, myoglobin, cytochromes, cytochrome oxidase

## Abstract

Diffuse optical spectroscopy (DOS) is a rapidly advancing non-invasive diagnostic technique to investigate biological tissue, based on probing the target object with optical radiation in the visible and/or near-infrared wavelength range and detecting the diffusely scattered light from the tissue. The signals obtained through DOS provide extensive information about the biochemical composition of tissues due to the presence of light-absorbing compounds known as chromophores. To date, DOS is widely employed to detect major chromophores such as deoxygenated (Hb) and oxygenated (HbO_2_) hemoglobin, water, lipids, and melanin. The concentrations of Hb and HbO_2_ in biological tissues are highly significant in clinical research, as they offer valuable insights into tissue oxygenation status and enable the detection of hypoxia. However, biological tissues also contain less-studied chromophores — minor chromophores — which also contribute to the overall absorption spectrum. These include various globins, such as methemoglobin, carboxyhemoglobin, and myoglobin, as well as cytochromes and cytochrome *c* oxidase. Identifying minor chromophores using DOS is challenging due to their relatively low absorption contributions compared to major chromophores, as well as the limited understanding of their specific absorption spectra. Nevertheless, the simultaneous detection of both major and minor chromophores could provide a comprehensive understanding of metabolic processes within vascular, intracellular, and mitochondrial compartments of tissues. This would substantially expand the potential applications of DOS in both research and clinical studies. In this review we examine literature sources that explore the investigation of minor chromophores in biological tissues by DOS, discuss the role of major chromophores, and evaluate the potential for simultaneous detection of both major and minor chromophores with DOS.

## Introduction

Optical methods for measuring physiological parameters of biological tissues are widely used in fundamental and clinical research due to their noninvasiveness, relatively low cost, compact equipment, and high information content. Tissue composition can be analyzed using various optical spectroscopic techniques, including Raman spectroscopy [1], elastic scattering (optical diffusion methods) [2, 3], and fluorescence methods [4]. While Raman spectroscopy and fluorescence techniques offer valuable insights, the detected signal levels in these methods are relatively low, which limits the depth of investigation. In contrast, optical diffusion methods offer significantly higher signal levels, enabling much greater penetration depths.

Diffuse optical spectroscopy (DOS) is a non-invasive diagnostic technique that allows for the assessment and monitoring of the biochemical state of tissues. DOS is widely employed for investigating brain hemodynamics (fNIRS) [5, 6], diagnosing and tracking malignant tumors [7–10] along with their microenvironment [11], evaluating scar severity and keloid treatment responses [12], early detection of pressure ulcers [13], assessing burn wound status [14], and various other applications. Additionally, it is employed for non-biomedical purposes, such as determining meat freshness [15] and conducting soil analysis [16]. DOS is based on probing the object with broadband optical and/or near-infrared radiation, recording the diffusely scattered light from the tissue, and solving the inverse problem of reconstructing the concentrations of tissue chromophores [17, 18]. The signal recorded by DOS carries information about both scattering, which is related to the tissue’s microstructure, and absorption, which reflects its biochemical composition [19]. The reconstruction of chromophore concentrations is possible due to the distinct partial absorption spectra of these compounds.

The accuracy of reconstructing chromophore concentrations is affected by numerous factors. For instance, distinguishing between chromophores with similar absorption spectra, such as hemoglobin and myoglobin, poses a significant challenge. Additionally, isolating the absorption contribution of a minor chromophore with a low concentration from that of a chromophore with a high absorption coefficient is challenging. Traditional models of light propagation in tissues, based on solving the radiative transfer equation in the diffusion approximation, have significant limitations for closely spaced sources and detectors and/or in cases of high absorption coefficients. This urge researches to create refined models. Furthermore, literature data on the absorption spectra of various chromophores often exhibit significant variations, which may reflect high sample variability and, ultimately, reduce the accuracy of reconstruction. Therefore, researchers often include only major chromophores, oxy- and deoxyhemoglobin (HbO_2_ and Hb), in the inverse problem of DOS, as they contribute the most to light absorption. However, data on the concentration of these chromophores alone enable the investigation of important physiological parameters, including tissue oxygenation and blood filling. Less commonly, studies assess water content to evaluate edema [20] or melanin content for melanoma diagnosis [21]. Studies on minor chromophores, such as methemoglobin (MetHb), myoglobin, carboxyhemoglobin (HbCO), cytochromes, and cytochrome *c* oxidase using DOS, are far less prevalent in the literature, despite their potential importance in addressing various biomedical challenges.

The difficulty in assessing the content of minor chromophores in tissue arises from their low concentrations, which result in minimal contributions to the overall absorption. Their detection requires highly sensitive measurements of tissue diffuse reflectance spectra, preferably over a wide wavelength range, as well as their inclusion in the diffuse scattering model. Moreover, available data on the partial absorption spectra of these chromophores are often limited in accuracy and the wavelength range covered by laboratory measurements.

In this review, literary sources containing information on minor chromophores of biological tissues were analyzed. Additionally, this paper provides data on major chromophores in biological tissues (water, lipids, melanin, and hemoglobin), as well as the possibility of their simultaneous study with minor chromophores. Special attention is given to studies that consider the measurement of minor chromophore concentrations using DOS. Also, brief information on DOS technology is also provided, with a focus on its potential for detecting minor chromophores.

The literature search was conducted using the PubMed, Scopus, and RSCI databases employing the following key words: absorption, bilirubin, chromophores, cytochrome *c*, diffuse reflectance spectroscopy, melanin, methemoglobin, near-infrared spectroscopy, oxygen saturation, scattering.

## Diagnosis of biological tissues using diffuse optical spectroscopy

There are various configurations of sources irradiating the tissue and detectors recording radiation diffusely scattered by the tissue for DOS registration. These configurations include transmission, reflection, circular and arbitrary arrangements. Transmission, circular and arbitrary configurations are typically used for specific locations where the study is conducted, such as the breast [22], hand joints [23], feet [24], etc. Such configurations allow for the collection of absorption data from large tissue volumes, including deeply located structures such as tumors and joints. A large depth of light penetration, up to several centimeters, can only be achieved within the therapeutic window of tissue transparency (700–1000 nm), where tissue absorption is minimal. However, within this range, the number of tissue chromophores contributing significantly to absorption is limited primarily to hemoglobin, water, and lipids. The absorption contributions of minor chromophores are challenging to isolate due to their low concentrations and the absence of pronounced peaks in the absorption spectrum.

**Figure 1.**
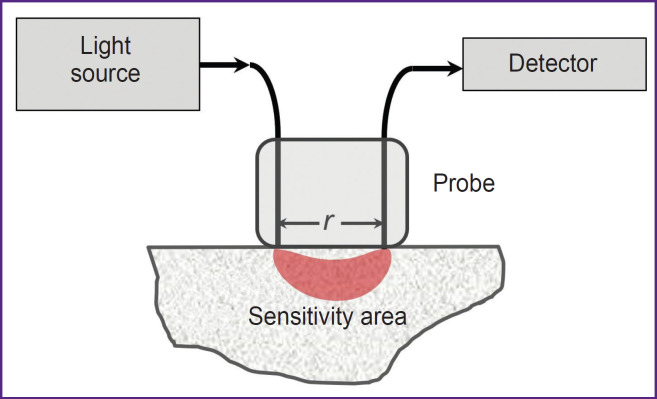
Diffuse optical spectroscopy in reflective configuration

In DOS systems with a reflective configuration, known as diffuse reflectance spectroscopy, a contact fiberoptic probe with one emitting and one receiving fiber is most commonly used (Figure 1). The distance between the source and detector can be adjusted based on the desired signal level and investigation depth. For example, in DOS studies using the visible wavelength range, the distance between the source and detector is typically limited to 5 mm due to strong light attenuation in tissues. Nevertheless, the visible wavelength range is particularly valuable for detecting minor tissue chromophores. More complex configurations with multiple sources and detectors can also be used to obtain spectroscopic data at different depths [25] or to mitigate the influence of instrumental functions, such as ratiometric approach or self-calibration method [19, 26].

When studying biological tissues using the DOS method, the multiple scattered diffuse component of light is recorded. The attenuation of this component depending on the source-detector distance *r* follows an exponential law with the extinction coefficient μ_*eff*_, which is determined by the absorption coefficient μ_*a*_ and the transport scattering coefficient μ*’_s_* as follows:


(1) $$\mu_{eff}=\sqrt{3\mu_{a}(\mu_{a}+\mu_{s}^{'})}.$$

The most common analytical model for describing diffusely scattered light is the solution to the radiative transfer equation in the diffusion approximation for a semi-infinite homogeneous scattering and absorbing medium, considering the directionality of the radiation source [27]. Advanced models also account for factors such as the detector fiber’s acceptance angle [28] or the characteristics of the scattering phase function [29], which are particularly significant when the source and detector overlap spatially.

The extinction coefficient for a homogeneous scattering and absorbing tissue can be estimated by measuring the intensity of diffusely scattered light at two source-detector distances. It is also acceptable to use measurements with a single source–detector distance, but this requires calibration of the DOS system on a model medium (tissue phantoms).

The absorption coefficient μ_*a*_, included in the extinction coefficient (1), can be expressed as a weighted sum of the absorption spectra of endogenous chromophores in the tissue [30]:


(2) $$\mu_{a}(\lambda )=\Sigma_{i}C_{i}\mu_{a}^{i}(\lambda ),$$

where *C_i_* is the concentration of the *i*-th chromophore, and $\mu_{a}^{i}(\lambda )$ is the absorption coefficient of the *i*-th chromophore at a given wavelength λ. This equation implies that the maximum number of chromophores that can be reconstructed from DOS measurements corresponds to the number of spectral lines at which the measurement is performed. However, even with a large number of spectral lines achieved using broadband source and a spectrometer, the reconstruction of a large number of chromophores is not guaranteed. One reason for this limitation is that diffuse scattering depends on both the absorption and the scattering coefficient, the latter of which is also unknown.

Hardware-based separation of scattering and absorption coefficients is possible using time-domain or frequency-domain methods [31–33]. These methods are particularly effective for large source–detector distances (several centimeters), where photons undergo sufficient scattering events to measure their time delay or a phase shift of a photon density wave. However, for the visible wavelength range, where source-detector distances are small, implementing these methods requires extremely high modulation frequencies (several gigahertz), posing significant technical challenges.

The SFDI method (spatial frequency domain imaging) represents a more promising approach for separating the absorption coefficients μ_*a*_ and μ*’_s_* in both visible and NIR wavelength ranges. However, as with the time-domain or frequency-domain approach, its implementation requires multiple light sources at different wavelengths and an image configurator, typically a specialized digital light processing matrix. Thus, SFDI is technically complex, costly and requires significant data acquisition time to acquire at least three images for each spatial frequency at each wavelength.

In most cases, the scattering spectrum used to solve the inverse problem in DOS is either derived from literature data specific to the tissue type or simplified by parameterizing it with a few parameters, assuming its monotonic decline as the wavelength increases. A comprehensive parametric formula for the transport scattering coefficient spectrum includes two components: Rayleigh scattering, which exhibits a –4^th^ power dependence on wavelength, describing scattering by particles smaller than the wavelength, and a component characterizing scattering by large particles with a power exponent *b*<4 [18, 34], which depends on the average size of these particles:


(3) $$\mu_{s}^{'}(\lambda )=a\left[f{\left(\frac{\lambda }{\lambda_{0}}\right)}^{-4}+(1-f){\left(\frac{\lambda }{\lambda_{0}}\right)}^{-b}\right],$$

where *a* is the reduced scattering coefficient at λ_0_=50 nm, *f* is the fraction of Rayleigh scattering. It is assumed that Rayleigh scattering dominates in the visible range, while Mie scattering (scattering by large particles) prevails in the near-infrared [19]. Thereby, for the visible range, only the Rayleigh scattering component is often used, while for the near-infrared range the scattering spectrum is either assumed constant or weakly depends on wavelength.

When implementing DOS using broadband radiation and a spectrometer, it is preferable to use as wide a spectrum range as possible to account for more chromophores. The inclusion of more absorption peaks from different chromophores within the measured spectrum enhances the accuracy of concentration determination. In practice, the spectral range of DOS studies is usually limited to 450–1000 nm. In the shortwavelength range, the limitation is due to the high absorption of chromophores such as melanin and hemoglobin, while the long-wavelength range is limited by the sensitivity of silicon detectors. For registration in the NIR-II range, spectrometers with a different type of matrix, such as InGaAs, are used. Although more expensive, these systems provide more reliable data on lipid and water concentrations [35].

Thus, the inverse problem of DOS — the reconstruction of chromophore concentrations *C_i_* from the measured diffuse spectrum — can be solved simultaneously with determining the scattering spectrum parameters *a*, *f*, *b*, included in equation (3) [36]. This approach is the simplest method for estimating the concentrations of minor chromophores over a wide wavelength range (visible to near-infrared). While the use of SFDI technique can improve measurement accuracy by independently estimating μ_*a*_ and μ*’_s_* at different wavelengths, but this method is more technically demanding.

## Chromophores of biological tissues

There are many different light-absorbing chromophores in human and animal tissues [37–70]. The most studied include deoxygenated and oxygenated hemoglobin, water, and lipids [37, 38]. In the visible and near-infrared wavelength ranges, where DOS measurements are typically conducted, these chromophores contribute the most to light absorption (2), and, accordingly, are well detected by DOS (see the Table).

**Table:** Endogenous chromophores of biological tissues: absorption peaks, biological role, and applications in DOS studies

Chromophore	Absorption peaks (nm)	Biological role	Known applications in DOS studies
***Major chromophores***			
Hemoglobin (oxyhemoglobin — HbO_2_, deoxyhemoglobin — Hb)	Hb: 274, 436, 556, 758, 934, 1160, 1540 [40] HbO_2_: 276, 342, 414, 542, 576, 938, 1511 [40]	Hemoglobin is the main protein of blood, responsible for oxygen transport. Hemoglobin can be saturated with oxygen molecules — oxyhemoglobin (HbO_2_), or desaturated with oxygen molecules — deoxyhemoglobin (Hb). HbO_2_ and Hb are the two main chromophores of blood. The hemoglobin molecule is composed of four subunit polypeptide chains and four heme groups. Heme is a porphyrin ring complexed with ferrous iron Fe^2+^ and protoporphyrin IX. The porphyrin ring in the heme contains a large number of conjugated double bonds, which allows the molecule to absorb light in the visible part of the spectrum	Determination of blood oxygen saturation (StO_2_=HbO_2_/THb) and total hemoglobin concentration (THb=HbO_2_+Hb) is the most common application of diffuse optical methods [30] The obtained StO_2_ values are used to assess tissue hypoxia [37] and tumor angiogenesis [41] DOS is used to monitor StO_2_ in tumor tissues during chemotherapy [8], radiation therapy [11], and photodynamic therapy [42]
Water	972, 1192, 1453, 1930 [43, 44]	Water is the natural medium for all biochemical reactions, participates in hydrolysis and redox reactions, forms hydration shells around large biomolecules (bound water). Bound water plays a critical role in stabilizing the structure of biomolecules, such as proteins and nucleic acids, by maintaining their three-dimensional conformations and preventing denaturation. Also, water plays a central role in edema, as its abnormal accumulation in interstitial spaces leads to tissue swelling	Malignant breast tumors contain more water and fewer lipids, allowing the use of the water/ lipid ratio to determine the resection margin using DOS in the near-infrared [9] Tumor tissues have an increased content of bound water, which allows healthy and tumor tissues to be distinguished using DOS in the VIS-NIR range [45] The DOS method in the range of 400–1000 nm with a step of 0.5 mm was used to monitor the dynamics of edema [46]
Lipids	760, 930, 1040, 1211, 1392, 1413 [47]	Lipids are present in significant amounts in adipose tissues, including subcutaneous fat and visceral fat that surrounds organs. Lipids are the main component of the cell membrane	The use of the water/lipid ratio for determining tumor resection margins, as well as for detecting benign neoplasms and cysts using DOS in the near-infrared range [48]
Melanin	335, monotonic decrease in μ_*a*_ with increasing wavelength [49]	Endogenous pigment. Plays a major role in skin homeostasis, providing absorption of harmful UV-radiation in the range of 320 to 400 nm. There are two types of melanin: eumelanin is black-brown in color and pheomelanin is red-yellow in color	DOS has been employed *in vivo* to measure melanin levels in tissues for the purpose of diagnosing melanoma [50–52]
***Minor chromophores***			
Bilirubin	453, 467 [53, 54]	Unconjugated (free) bilirubin is mainly formed in the spleen as a result of hemolysis of red blood cells. Since unconjugated bilirubin is insoluble in water, it binds to albumin in the bloodstream for transport to the liver, where it is conjugated with glucuronic acid to form water-soluble conjugated bilirubin, which can then be excreted in bile	DOS in the range of 400–700 nm was used to study bilirubin content, HbO_2_ and Hb, as well as blood oxygenation in newborns [55] Including bile in the reconstruction model significantly decreased the mismatch between the model and the experimentally measured absorption spectra of healthy liver tissues [43] DOS revealed that liver tumor tissues contain five times less bile compared to healthy tissues [43]
Carbaminohemoglobin (HbCO_2_)		HbCO_2_ is a form of hemoglobin bound to carbon dioxide CO_2_ through the α-amino groups of each of the four protein chains of globin	The absorption spectra of HbCO_2_ and Hb are very close and practically overlap, making it difficult to determine HbCO_2_ concentration in tissues using non-invasive optical methods
Carboxyhemoglobin (HbCO)	538, 569 [56]	Formed when hemoglobin binds to carbon monoxide. HbCO level increases with carbon monoxide (CO) exposure, since CO has 200– 300 times greater affinity for hemoglobin than oxygen	Reconstruction of HbCO in DOS studies is extremely difficult, as the absorption spectrum of HbCO is not well studied. Only two peaks in the visible range, close to the absorption peaks of HbO_2_ and MbO_2_, have been detected
Collagen	930, 1050, 1200, 1500, 1700 [57]	Collagen is a critical structural protein in biological tissues, providing mechanical strength and elasticity to the extracellular matrix. Collagen is the main component of connective tissue, forming the basis for tendons, cartilage, bone tissue, etc.	DOS in the SWIR-II range (2.1-2.4 μm) was used to study collagen content in cartilage *ex vivo* [58] In the range of 635-1060 nm, a high level of collagen in breast tissue was shown to correlate with tissue density and, accordingly, with the risk of breast cancer [59]
Methemoglobin (MetHb)	405, 500, 631 [60]	MetHb is an oxidized form of hemoglobin in which the iron in the heme group is in the ferric (Fe^3+^) state rather than the ferrous (Fe^2+^) state, rendering it incapable of binding oxygen. MetHb accumulation, often due to oxidative stress or genetic disorders, can alter tissue optical properties and serve as a biomarker for conditions such as ischemia, hemorrhage, or methemoglobinemia [61, 62]	Inclusion of MetHb in the reconstruction model significantly reduced the discrepancy between the model and measured absorption spectra of breast tissues [63] DOS in the VIS-NIR range (400–1000 nm) with a step of 10 nm registered an increase in MetHb levels due to changes in skin hemodynamics during burns [14]
Metmyoglobin	504, 633 [64]	Metmyoglobin is oxidized form of myoglobin	DOS was used to assess the ratio of metmyoglobin and oxygenation in pork and beef to evaluate product quality [15]
Myoglobin (oxygenated, MbO_2_, deoxygenated, Mb)	Mb: 561 [56] MbO_2_: 554, 584 [56]	Myoglobin is a small oxygen- and ferrous-binding protein found primarily in muscle tissues, where it facilitates oxygen storage and diffusion to mitochondria. Unlike hemoglobin, myoglobin contains only one heme and one polypeptide chain	DOS in the visible and near-infrared range (540–800 nm) introduced a muscle oxygenation index, considering the content of MbO_2_ and Mb in the blood [65]
Cytochrome *c* in reduced (redCyto *c*) and oxidized (oxCyto *c*) form	redCyto *c*: 414, 519, 550 [66] oxCyto *c*: 408, 530 [66]	Cytochrome *c* is a small heme protein located in the inner mitochondrial membrane, playing a critical role in the electron transport chain and cellular respiration. Due to its ability to alternate between oxidized (oxCyto *c*) and reduced (redCyto *c*) states, cytochrome *c* can provide valuable information about mitochondrial oxygenation levels [37]	Research on cytochrome *c* using DOS is limited in the literature primarily because of the spectral overlap between cytochrome *c* and hemoglobin. However, an optical “window” between 540 and 585 nm has been identified, enabling the detection of myoglobin and cytochrome *c* without significant interference from HbO_2_ and Hb [67]
Cytochrome *c* oxidase in reduced (redCCO) and oxidized (oxCCO) form	redCCO: 446, 616 [68] oxCCO: 421. 600 [68]	Cytochrome *c* oxidase is a key enzyme in the mitochondrial electron transport chain, responsible for the final step of cellular respiration by transferring electrons to oxygen, forming water. Can be found in oxidized and reduced states (oxCCO/redCCO)	Incorporating cytochrome *c* oxidase into the light propagation model for tissues enhanced the accuracy of reconstructing the concentrations of other chromophores [69] Additionally, DOS in the 650–1000 nm range enabled simultaneous monitoring of total hemoglobin, HbO_2_, Hb, oxCCO and redCCO in muscle tissues of model animals during cyanide poisoning [70]

The absorption of DNA and most polypeptides are predominantly in the ultraviolet (UV) spectral range. In particular, aromatic amino acids — tryptophan, tyrosine, and phenylalanine — absorb in the UV-range, resulting in most proteins absorbing light at a wavelength of 280 nm [39]. Thus, DNA and polypeptides don’t contribute significantly to absorption in the visible and near-infrared ranges and are not detectable by DOS within these spectral regions.

**Figure 2.**
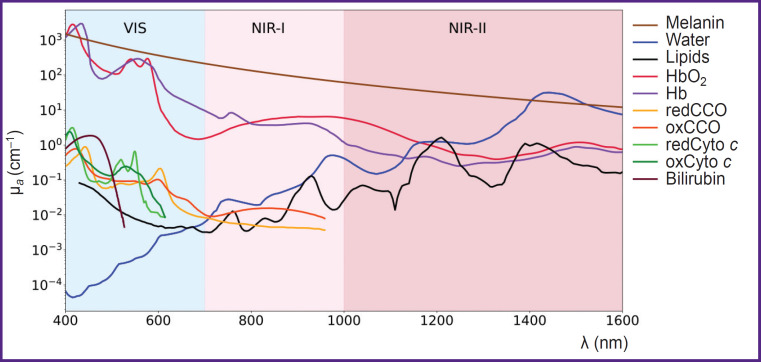
Absorption spectra of endogenous chromophores (adapted from [40]) HbO_2_ and Hb — oxygenated and deoxygenated hemoglobin, redCCO and oxCCO — cytochrome *c* oxidase in reduced and oxidized forms, redCyto *c* and oxCyto *c* — cytochrome *c* in reduced and oxidized forms

In Figure 2 the spectra of major chromophores, HbO_2_, Hb, water, melanin, lipids, and some minor chromophores, cytochrome *c* oxidase, cytochrome *c*, bilirubin, are presented. As shown in the figure, the absorption spectra of these chromophores exhibit distinct features, enabling the potential quantification of their concentrations based on the DOS measurements [13].

## Major chromophores of biological tissues

### Water

Water is one of the most abundant biological compound in the human body, playing a vital role in hydrolysis and redox reactions. It constitutes 60 to 80% of a living cell’s mass and is one of the primary chromophores in biological tissues. While the absorption coefficient of water is insignificant in the visible wavelength range, exhibits several absorption peaks in the near-infrared range [7] (see Figure 2). The water/ lipid ratio in breast cancer tissue can provide important information about tumor structure. For instance, studies have shown that malignant breast tumors exhibit higher water content and lower lipid levels compared to normal tissue [71]. In the study by Veluponnar et al. [9], it was shown that absorption in the near-infrared region, related to the fat/water ratio in tissue, allows the use of DOS to assess the resection margin during breast cancer surgery.

**Figure 3.**
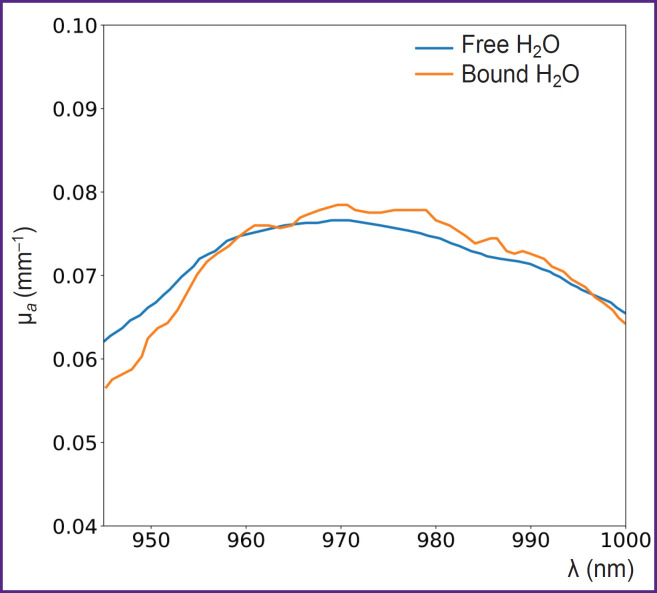
Absorption spectra of pure free water and bound water (adapted from [73])

Another application of DOS is the assessment of bound and free water fractions in tissues. Bound water, which forms hydration shells around biopolymers through electrostatic interactions [72], is essential for stabilizing intracellular macromolecules and membranes, as well as facilitating the diffusion of substances across membranes. The fraction of bound water introduces small changes in the shape of the water absorption spectrum within tissues (Figure 3), shifting the water absorption maximum by several nanometers [73]. This property has been exploited to distinguish malignant breast cancer tissues from normal tissues. For instance, Chung et al. [45] demonstrated by employing DOS in the visible to near-infrared (VIS-NIR) range, that breast tumor tissues contain significantly higher levels of bound water compared to healthy tissue.

### Lipids

In the human body, lipids are present in significant amounts within adipose tissues, including subcutaneous fat (located beneath the skin) and visceral fat (surrounding internal organs). For example, a dense layer of adipose tissue surrounds the breast. Additionally, lipids are the main component of the cell membrane. Like water, lipids are major absorbing chromophores in the near-infrared wavelength range [74]. The presence of lipids in a sample is characterized by a narrow absorption peak around 1211 nm [47] (see Figure 2).

Tissues with a high content of membrane organelles in cells (mitochondria, lysosomes) exhibit pronounced Rayleigh scattering due to the high density of lipid membranes, causing *b* and *f* in equation (3) to increase [75, 76].

The water/lipid ratio in breast tissues can be used not only for monitoring and resection of malignant tumors but also for detecting benign neoplasms and cysts [48].

It is important to note that the properties and functions of lipids directly depend on the fatty acids they contain. In turn, fatty acids differ in chain length and the presence of double bonds in their structure. Saturated, monounsaturated, and polyunsaturated fatty acids are distinguished. Typically, the subcutaneous adipose tissue of an adult human contains 21, 46, and 33% saturated, monounsaturated, and polyunsaturated fatty acids, respectively. Variations in this ratio lead to small changes in the lipid absorption spectrum. For example, a sample consisting of 42, 46, and 12% saturated, monounsaturated, and polyunsaturated fatty acids, respectively, shows an additional absorption peak at 1170 nm [47].

For confident separation of lipids and water, it is preferable to use the NIR-II spectral range, where the absorption peaks of water and lipids are well-separated (see Figure 2). In the NIR-I range, the absorption peaks of lipids (930 nm) and water (975 nm) overlap significantly, making their separation challenging [77, 78].

### Melanin

Melanin is an endogenous pigment, an insoluble high-molecular-weight polymer responsible for the coloration of eyes, hair, and skin. It is produced by melanocytes through a process called melanogenesis in cytoplasmic organelles known as melanosomes. In the epidermis, melanocytes transport melanosomes to keratinocytes through dendritic processes [79]. Melanin plays a crucial role in skin homeostasis, protecting tissues from UV radiation by absorbing and dissipating UV light in the range of 320 to 400 nm [80]. Melanin exists in different forms, such as eumelanin (brown/ black) and pheomelanin (red/yellow), contributing to the diversity of pigmentation in humans and other organisms.

Quantitative determination of melanin using noninvasive methods is important for clinical studies. Abnormal level of melanin is an indicator of various diseases. In particular, objective measurements of melanin content in tissues can aid in differentiating melanoma from benign pigmented neoplasms, as well as for assessing pigmented diseases and monitoring therapeutic responses [81].

Several studies demonstrated the use of DOS method for the *in vivo* quantitative assessment of melanin content in tissues for melanoma diagnosis [50–52]. For instance, DOS showed that eumelanin content increases during the progression from dysplastic nevus to invasive melanoma, while pheomelanin content decreases [51].

It is important to note that the absorption spectrum of melanin exhibits a monotonic decrease as wavelength increases, similar to the behavior of the transport scattering coefficient. This similarity complicates the reconstruction of melanin concentration in DOS systems that do not employ hardware-based separation of scattering and absorption coefficients.

### Hemoglobin

Hemoglobin is a complex protein composed of four polypeptide chains, each associated with a heme group. The porphyrin ring in the heme group contains an extensive system of conjugated double bonds, which gives it the ability to absorb light in the visible part of the spectrum. The protein component of the molecule, globin, consists of two α- and two β-subunits. The heme, a complex of protoporphyrin IX with an iron atom Fe^2+^. These iron atoms in the Fe^2+^ state can bind to oxygen [82].

Hemoglobin exists in two primary states based on its oxygen binding: oxyhemoglobin (HbO_2_), which is saturated with oxygen molecules, and deoxyhemoglobin (Hb), which is unsaturated and lacks bound oxygen. HbO_2_ and Hb are the major chromophores of blood, with their absorption spectra shown in Figure 2. Determination of blood oxygenation is the most common application of optical diffusion methods. For example, pulse oximetry assesses arterial blood oxygenation by exploiting differences in absorption spectra in the visible range (500–600 nm) in reflective configuration (e.g., fitness bracelets and smartwatches), and in the near-infrared range (700–900 nm) in pulse oximeters with transmission or reflective configurations [83]. In contrast to pulse oximetry, DOS delivers average tissue oxygenation values [30, 84–86], which differ fundamentally from the measurements acquired through pulse oximetry.

Hemoglobin (Hb) and oxyhemoglobin (HbO_2_) are of significant interest in clinical research because they provide critical information about total hemoglobin concentration (THb=HbO_2_+Hb) and blood oxygenation level (StO_2_=HbO_2_/THb) [30]. Experimentally measured StO_2_ values are utilized to evaluate tissue hypoxia [37] and study tumor angiogenesis [41]. Hypoxia causes tumor resistance to standard therapy and promotes the development of an aggressive phenotype [87]. Therefore, timely assessment of tumor oxygenation can play a crucial role in optimizing and adjusting treatment strategies. The use of DOS for monitoring tumor tissue oxygenation has been demonstrated during chemotherapy [8], radiation therapy [11], and photodynamic therapy [42].

## Minor chromophores of biological tissues

**Figure 4.**
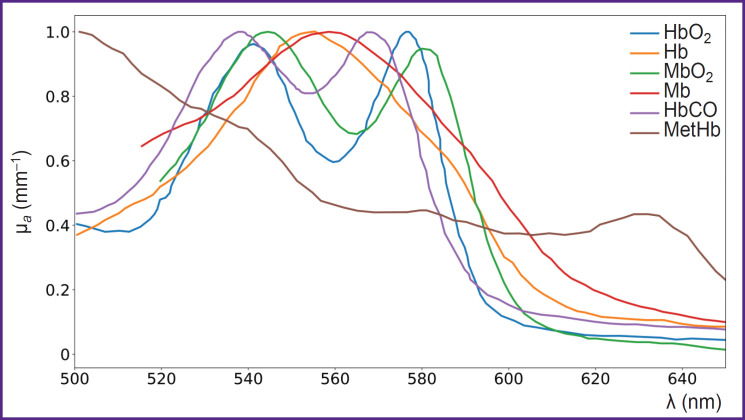
Partial absorption spectra (adapted from [56, 89]) HbO_2_ and Hb — oxygenated and deoxygenated hemoglobin, MbO_2_ and Mb — oxygenated and deoxygenated myoglobin, HbCO — carboxyhemoglobin, MetHb — methemoglobin. Data normalized to maximum values

In addition to the major forms of hemoglobin, human blood contains derivatives (MetHb, HbCO, ect.). Monitoring these derivative forms is crucial for studying various pathologies, such as toxic exposures, metabolic disorders, and conditions affecting oxygen transport [88]. It is important to note that the absorption spectra of different forms of hemoglobin, can vary significantly [56, 89, 90]. These distinct spectral features enable their differentiation and quantification using DOS (Figure 4).

### Carboxyhemoglobin

Carboxyhemoglobin (HbCO) is formed when hemoglobin binds to carbon monoxide (CO) instead of oxygen, reducing its oxygen-carrying capacity. The level of HbCO increases with CO exposure, as CO has 200–300 times greater affinity for hemoglobin than oxygen [91]. The HbCO content in the blood is an important physiological indicator in carbon monoxide poisoning. Additionally, early studies using electrocardiography noted that the presence of CO in inhaled air during physical activity can have adverse effects on patients with cardiovascular diseases, even at low concentrations [92]. Therefore, monitoring HbCO concentration in tissues may be of interest in clinical studies.

The absorption spectrum of HbCO is not well characterized, with only two peaks in the visible range, close to the absorption peaks of HbO_2_ and MbO_2_ [56] (see Figure 4), complicating HbCO reconstruction using DOS.

### Carbaminohemoglobin

Carbaminohemoglobin (also known as carbhemoglobin) (HbCO_2_) is a form of hemoglobin that binds to carbon dioxide (CO_2_) for transport from tissues to the lungs, where it is exhaled. Unlike HbO_2_, which carries oxygen, HbCO_2_ forms when CO_2_ binds to the amino groups of the globin chains (not the heme iron). Dervieux et al. [93] isolated HbCO_2_ and Hb from human blood and measured their absorption spectra in two ranges — 235–600 nm and 600–1000 nm. The results showed that the absorption spectra of HbCO_2_ and Hb are very close and practically overlap. Therefore, it is difficult to determine HbCO_2_ concentration in tissues using non-invasive optical methods.

### Methemoglobin

Methemoglobin (MetHb) is an oxidized form of hemoglobin in which the iron in the heme group is in the ferric Fe^3+^ state instead of the normal ferrous Fe^2+^ state. The enzyme cytochrome *b*_5_ reductase (also known as NADH-cytochrome *b*_5_ reductase) plays a critical role in reducing MetHb back to its functional form, Hb, by converting the ferric iron Fe^3+^ in MetHb to Fe^2+^. Under normal physiological conditions, the concentration of MetHb in human blood is very low, typically around 1–2% of total hemoglobin.

MetHb cannot bind oxygen and, as a result, it is incapable of transporting oxygen to tissues. Elevated levels of MetHb reduce the overall oxygen-carrying capacity of the blood, leading to tissue hypoxia and symptoms such as cyanosis (bluish discoloration of the skin) and shortness of breath, despite a possible normal level of total hemoglobin [61, 62]. MetHb levels are traditionally assessed using biochemical methods, such as high-performance liquid chromatography or electrophoresis [94]. Elevated MetHb levels have been found in patients with sepsis, infants with severe metabolic acidosis (blue baby syndrome), and individuals with rare congenital metabolic abnormalities, such as glucose-6-phosphate dehydrogenase deficiency [95].

Since the nitrite ion NO^2–^ oxidizes Fe^2+^ to Fe^3+^, MetHb formation in the body can occur as a result of nitrate poisoning and acquired methemoglobinemia. It has been noted that methemoglobinemia can be acquired through exposure to various drugs (such as benzocaine, dapsone, sulfonamides, and nitrate derivatives) or other chemical compounds that are strong oxidizers [61]. Therefore, MetHb content in biological tissue is an important clinical indicator.

**Figure 5.**
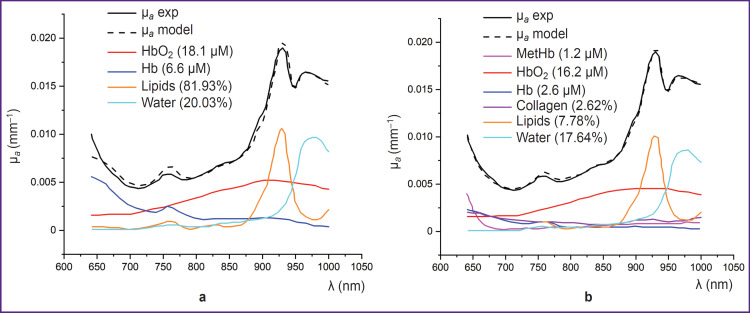
Absorption spectrum of breast tumor tissue, expressed as the sum of the absorption spectra of major chromophores (a) and major chromophores with the addition of minor ones (b) (adapted from [63]) HbO_2_ and Hb — oxygenated and deoxygenated hemoglobin, MetHb — methemoglobin

MetHb has an absorption peak of about 630 nm, making it noticeable against other globins [95] (see Figure 4). In the study using DOS in the NIR range (640–1000 nm), Vasudevan et al. [63] demonstrated that including MetHb in the tissue chromophore absorption spectrum — alongside HbO_2_, Hb, water, lipids, and collagen — significantly reduced the discrepancy between the modeled and experimentally measured absorption spectra (Figure 5). Additionally, this study found that MetHb concentration in tumor tissues is higher than in normal breast tissues. Since MetHb concentration in healthy tissues is extremely low, it can serve as a potential oncological marker.

Another application of DOS is the monitoring of burn wounds. Using DOS in the VIS-NIR range (400– 1000 nm) with a 10 nm resolution, researchers observed an increase in MetHb levels associated with changes in skin hemodynamics during burn injuries [14]. In this study, Khatun et al. [96] developed a method for the joint quantitative assessment of melanin, HbO_2_, Hb, and MetHb in rat tissue models with methemoglobinemia. This method was then applied to classify burns by severity and assess burn depth in rats.

### Myoglobin

Myoglobin is a small, oxygen-binding protein primarily found in muscle tissues, where it plays a key role in oxygen storage and diffusion to mitochondria for cellular respiration. Structurally, myoglobin consists of a single polypeptide chain and a heme group. Its primary function is to reversibly bind oxygen and facilitate oxygen diffusion from blood capillaries into muscle mitochondria [97]. Additionally, myoglobin serves as a biomarker for muscle damage, as its release into the bloodstream is indicative of conditions such as rhabdomyolysis or myocardial infarction. A major focus in preclinical and clinical studies of myoglobin is the quantitative assessment of myoglobin oxygen saturation (sO_2_-Mb) [98].

**Figure 6.**
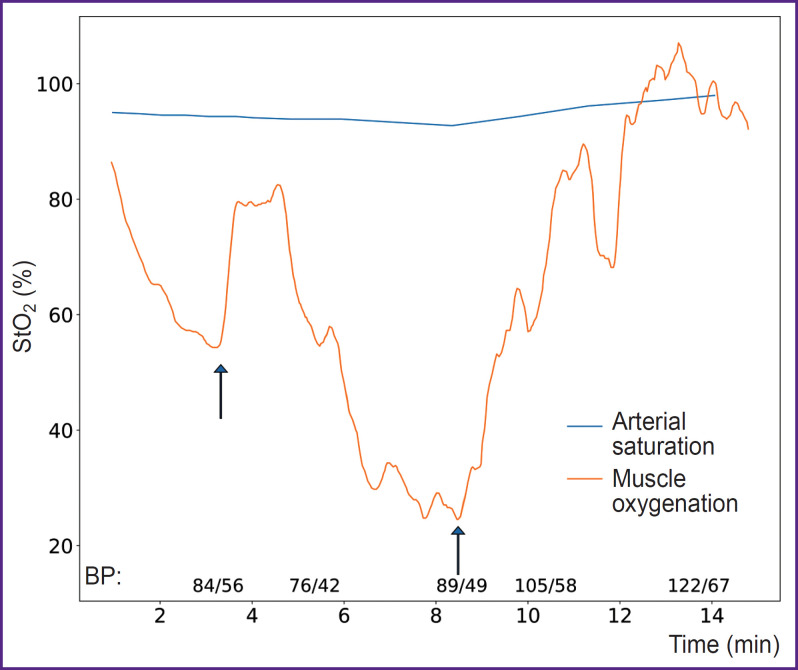
Muscle oxygenation provides information about tissue perfusion (adapted from [65]) A patient with trauma was administered saline solution for t=2 and 8 min (indicated by arrows), leading to an increase in blood pressure (BP) and a corresponding increase in muscle oxygenation. Arterial saturation, measured using pulse oximetry, was not sensitive to changes in tissue perfusion, unlike the muscle oxygenation index

In the study using DOS in the VIS-NIR range (540–800 nm), Arakaki et al. [65] introduced a muscle oxygenation index, which represents the percentage of oxygenated myoglobin and hemoglobin relative to the total amount of myoglobin and hemoglobin in the tissue. The results demonstrated that measuring the oxygenation of both hemoglobin and myoglobin together provides a more accurate assessment of muscle oxygenation compared to measuring only arterial components (Figure 6).

### Metmyoglobin

Metmyoglobin is the oxidized form of myoglobin. In living muscle tissue, the concentration of metmyoglobin is extremely low due to the activity of the enzyme metmyoglobin reductase. This enzyme, in the presence of the cofactor NADH and the coenzyme cytochrome *b*_5_, reduces the ferric iron (Fe^3+^) in the heme group of metmyoglobin back to ferrous iron Fe^2+^ restoring it to functional myoglobin. This reduction process is essential for maintaining the oxygen-binding capacity of myoglobin and ensuring efficient oxygen storage and diffusion in muscle tissues.

In meat science, the accumulation of metmyoglobin is associated with the browning of meat, as it alters the pigment’s color from red to brown [99]. To extend the shelf life of the meat product, it is extremely important to detect the formation and accumulation of metmyoglobin, as well as to measure meat oxygenation. Like methemoglobin, metmyoglobin has a distinct absorption spectrum, which allows its detection and quantification using spectroscopic techniques. In particular, DOS was used to assess the ratio of metmyoglobin and oxygenation in pork and beef [15].

### Bilirubin

Heme is released when red blood cells reach the end of their lifespan and are broken down in the spleen. The enzyme heme oxygenase then catalyzes the conversion of heme into biliverdin, a green pigment. This reaction also produces a free iron ion Fe^3+^ and CO as byproducts. Biliverdin consists of four pyrrole rings connected in a linear chain. Biliverdin is then further reduced to free (unconjugated) bilirubin by the enzyme biliverdin reductase. Free bilirubin is poorly soluble in water and is transported by the bloodstream as a complex with the plasma protein albumin [39]. Each albumin molecule can bind two bilirubin molecules. With the bloodstream, unconjugated bilirubin is transported to the liver where it undergoes further metabolism in hepatocytes. Here, it is conjugated with glucuronic acid by the enzyme UDP-glucuronosyltransferase, forming bilirubin diglucuronide (also known as conjugated bilirubin). This conjugated form is water-soluble and insolubility in fats, unlike its unconjugated counterpart, and is excreted as a key component of bile into the small intestine [100].

Under conditions of increased hemolysis of red blood cells, impaired liver function, or blockage of the bile ducts, the level of bilirubin in the blood rises. Bilirubin acts as an antioxidant in mammalian tissues, helping to neutralize reactive oxygen species and protect cells from oxidative damage. However, when the concentration of bilirubin in the serum exceeds the binding capacity of serum albumin, it can lead to bilirubin intoxication. This condition results in the accumulation of free, unbound bilirubin in the blood, causing hyperbilirubinemia and potentially leading to jaundice, characterized by yellowing of the skin and eyes [100]. In most infants, hyperbilirubinemia is a normal and temporary condition, commonly referred to as physiological jaundice. However, in some infants, particularly premature babies, hyperbilirubinemia can result in the accumulation of bilirubin in brain tissue when high levels of unbound bilirubin cross the blood-brain barrier, leading to a condition known as kernicterus [101]. Therefore, it is recommended to carefully monitor bilirubin levels in serum during neonatal jaundice, especially in the first 24 h [102].

Thus, bilirubin is an important chromophore for assessing health status in neonatal jaundice, liver cirrhosis, and hepatitis. Traditionally, the levels of various forms of bilirubin in the blood are assessed using invasive biochemical methods. These methods involve drawing a blood sample and analyzing the serum to quantify bilirubin concentrations. Non-invasive alternatives, such as transcutaneous bilirubinometry or optical spectroscopy, are increasingly being explored to provide real-time, painless assessments of bilirubin levels [55].

The absorption maximum of bilirubin lies within the range of 400–500 nm (see Figure 2). In the study by Banerjee et al. [55], DOS in the range of 400–700 nm was employed to measure bilirubin content, HbO_2_, Hb, and blood oxygenation in 4668 newborns with ages ranging from 28 to 40 weeks. Similar indicators were measured using invasive biochemical methods. The values obtained by the two different methods had a high degree of correlation. In particular, for bilirubin *r*=0.88.

Another application of DOS is the monitoring of malignant tumors. Nachabé et al. [43] studied the absorption coefficient of bile in healthy and tumor tissues of the liver using DOS. The study used a setup with two spectrometers, covering the ranges of 400–1100 nm and 800–1700 nm. The light scattering model included major chromophores such as water, lipids, HbO_2_, and Hb. The results showed that including bile in the reconstruction model significantly reduced the discrepancy between the model and measured absorption spectra of healthy liver tissues. Additionally, the authors found that tumor tissues of the liver contain approximately five times less bile than healthy tissues, allowing the use of noninvasive methods for classifying healthy and cancerous tissues during ablation.

#### Cytochromes and cytochrome oxidase

As mentioned earlier, the concentrations of HbO_2_ and Hb allow for the assessment of blood filling and tissue oxygenation, while the registration of cytochromes and cytochrome oxidase allows for the monitoring of oxidative metabolism in tissue mitochondria [103].

Cytochromes are proteins with an iron-containing prosthetic group known as heme. The heme group features a system of conjugated double bonds, giving cytochromes their characteristic absorption peaks in the visible spectrum. Mitochondria contain three classes of cytochromes: *a*, *b*, and *c*, each with distinct absorption spectrum. For example, the absorption peak of cytochrome *a* is shifted to the long-wavelength region around 600 nm, cytochrome *b* around 560 nm, and cytochrome *c* around 550 nm [39].

**Figure 7.**
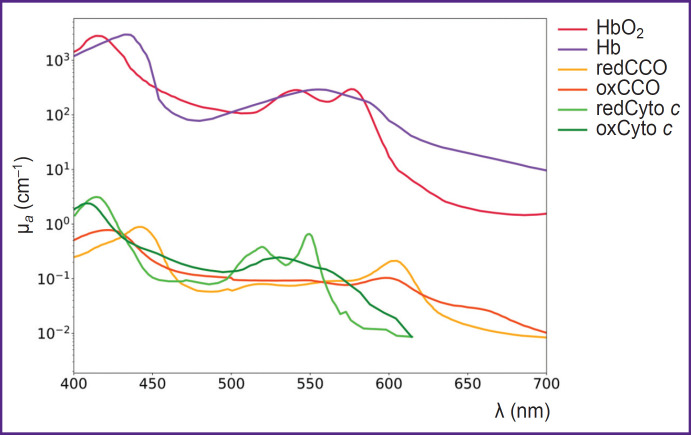
Absorption spectra of cytochrome *c* oxidase and cytochrome *c* (adapted from [40, 66, 68]) HbO_2_ and Hb — oxygenated and deoxygenated hemoglobin, redCCO and oxCCO — cytochrome *c* oxidase in reduced and oxidized forms, redCyto *c* and oxCyto *c* — cytochrome *c* in reduced and oxidized forms

Cytochrome *c* is a crucial component of the mitochondrial respiratory chain, playing a key role in electron transport during cellular respiration. Its ability to alternate between oxidized (oxCyto *c*) and reduced (redCyto *c*) states makes it a dynamic indicator of mitochondrial oxygenation levels [37]. In the study [104], it was demonstrated that the absorption spectra of oxidized cytochrome *c* (oxCyto *c*) and reduced cytochrome *c* (redCyto *c*) measured in pig heart tissues are distinct (Figure 7). Studies of cytochrome *c* using DOS are not widely covered in the literature due to the overlap of cytochrome and hemoglobin absorption spectra. However, an *in vivo* DOS study on pig heart identified an optical “window” from 540 to 585 nm, allowing for the detection of myoglobin and cytochrome *c* with minimal interference from HbO_2_ and Hb [67].

Among the components of the mitochondrial respiratory chain, cytochrome *c* oxidase has received the most attention in DOS studies. For example, including cytochrome *c* oxidase in the light propagation model in tissues improved the reconstruction quality of other chromophores [69].

Cytochrome *c* oxidase, also known as complex IV, is a key enzyme in the mitochondrial electron transport chain and belongs to the group of oxidoreductases. The complex is localized in the inner mitochondrial membrane and functions as a homodimer, with each monomer consisting of 13 subunits. Subunit I contains two heme groups: heme *a* and heme *a*_3_, as well as a copper ion Cu_B_. Heme *a*_3_ and Cu_B_ form a binuclear Fe– Cu center, which plays a critical role in the enzyme’s function. This center receives electrons from heme *a* and transfers them to molecular oxygen bound at the active site of heme *a*_3_. Subunit II contains two copper ions, which are bound to two cysteine residues via –SH groups, also forming a binuclear center Cu_A_.

Each of these centers can exist in oxidized or reduced states and has different absorption spectra. Despite the fact that the dominant chromophore in the nearinfrared range is the Cu_A_, center, other redox centers of cytochrome can also contribute to the formation of the resulting absorption spectrum of cytochrome *c* oxidase [104]. For example, it has been established that the fully oxidized binuclear center *a*_3_/Cu_B_ demonstrates an absorption peak at 655 nm.

Cytochrome *c* oxidase catalyzes the transfer of electrons from cytochrome *c* to molecular oxygen, reducing oxygen to water [105]. Simultaneous monitoring of the redox state of cytochrome *c* oxidase (oxCCO/ redCCO) and hemoglobin can provide additional information on hemodynamics, oxygenation, and tissue metabolism [104]. Using DOS in the range of 650 to 100 nm, Lee et al. [70] conducted joint registration of total hemoglobin, HbO_2_, and Hb, as well as oxCCO and redCCO in muscle tissues of model animals during cyanide poisoning. The results demonstrated that an optical window ranging approximately from 540 to 585 nm was identified in the pig heart in vivo, enabling the monitoring of myoglobin and cytochrome *c* without interference from hemoglobin oxygenation or blood volume variations.

It has also been shown that monitoring cytochrome *c* oxidase using DOS in the near-infrared correlates with other metabolic indicators, obtained through magnetic resonance spectroscopy or invasive methods, such as cerebral microdialysis [106, 107]. In particular, Tisdall et al. [106] studied the effect of hyperbaric oxygen therapy after traumatic brain injury (TBI). Since TBI is accompanied by impaired aerobic metabolism and mitochondrial dysfunction, the study monitored the concentration of oxidized cytochrome *c* oxidase using DOS in the nearinfrared range (650 and 980 nm). The results showed that changes in oxCCO concentration correlated with changes in brain tissue oxygenation (*r*=0.57, p=0.005).

One of the most interesting applications of DOS may be the joint registration of major and several minor chromophores. It has been established that the musclespecific oxygen-binding protein, myoglobin, localized in mitochondria, interacts with complex IV of the respiratory chain. This suggests that myoglobin may be a factor regulating mitochondrial respiration [108].

The joint registration of myoglobin, HbO_2_, oxygen saturation, and cytochrome *c* oxidase using DOS *in vivo* offers a comprehensive understanding of the physiological state of biological tissues. Arakaki et al. [109] determined the absorption spectra of heme *aa_3_* of cytochrome *c* oxidase, HbO_2_ and Hb, as well as MbO_2_ and Mb in the range of 600–850 nm *in vitro*. Then they applied the DOS method for simultaneous monitoring of major chromophores with cytochrome *c* oxidase and other minor chromophores in an *in vivo* experiment on rabbit forelimb muscle during ischemia. The study focused on the absorption spectrum of heme *aa_3_* cytochrome *c* oxidase. Assessment of the redox status of heme *aa_3_*, as well as oxygenation of hemoglobin and myoglobin *in vivo* was also conducted in the range of 600–850 nm. The results showed that during ischemia of the rabbit forelimb, hemoglobin desaturation occurs first, followed by myoglobin, and finally, the reduction of heme *aa_3_*. To solve the problem of overlapping spectra of the studied chromophores, the authors used the second derivative of the *in vitro* absorption spectrum obtained in the experiment, which provided betterdefined absorption peaks for Hb, Mb, oxССO*aa_3_*, and redCCO*aa_3_* compared to original spectra.

## Conclusion

The presence of light-absorbing compounds, known as chromophores, in biological tissues enables widespread use of DOS for the diagnosis and monitoring of various pathologies. Among the major chromophores of biological tissues are water, lipids, melanin, oxyand deoxyhemoglobin. Monitoring the water content in tissues can help detect edema, while the lipid/water ratio in tumors can assist in determining resection margins. Monitoring oxy- and deoxyhemoglobin allows to determine the degree of tissue oxygenation, detect hypoxia, and evaluate the effectiveness of antitumor therapy, among other applications. Additionally, melanin content in tissue serves as an important criterion for diagnosing melanoma stages.

In addition to major chromophores, biological tissues contain minor ones, such as bilirubin, various globins (carboxyhemoglobin, methemoglobin, myoglobin, etc.), as well as cytochromes and cytochrome *c* oxidase. Comprehensive study of major and minor tissue chromophores allows for simultaneous monitoring of metabolic processes in vascular, intracellular, and mitochondrial compartments. This approach is not only valuable in physiological studies of tissue metabolism but also for clinical applications, particularly in cases where oxygen supply to tissues is impaired. For example, assessment of the redox status of cytochrome *c* oxidase for monitoring therapy after traumatic brain injury, monitoring methemoglobin for assessing burn depth *in vivo*, assessment of myoglobin oxygenation in tissues for monitoring muscle metabolism after injury, and a number of other, currently not considered in the literature, applications. Furthermore, incorporating minor chromophores in the light scattering model in tissues improves the accuracy of reconstructing the concentrations of other chromophores from the signal recorded by DOS.
